# Do college students with future work self-salience demonstrate higher levels of career adaptability? From a dual perspective of teachers and students

**DOI:** 10.3389/fpsyg.2022.1011447

**Published:** 2022-09-15

**Authors:** Lei Lu, Qiuhong Jia

**Affiliations:** ^1^School of Business, Macau University of Science and Technology, Taipa, Macao SAR, China; ^2^College of Mechanical Engineering, Chongqing University of Technology, Chongqing, China

**Keywords:** future work self-salience, teacher support, career exploration, effective part-time behavior, career adaptability

## Abstract

Faced with tremendous employment pressure, how to enhance effective career exploration and career adaptability is crucial for college students’ career. This study uses self-assessed data from 840 undergraduate students at three time points to reveal the formation mechanism of career adaptability from a dual perspective of teacher support and students’ effective part-time behavior. In particular, the mediating role of career exploration is introduced based on self-regulation theory, and the moderating role of teacher support and students’ effective part-time work is introduced based on social cognitive career theory. The results show that (1) Future work self-salience positively influences career adaptability; (2) future work self-salience indirectly influences career adaptability through career exploration; (3) both teacher support and students’ effective part-time behavior positively moderate the indirect relationship between future work self-salience and career adaptability through career exploration. This study attempts to provide practical guidance for college graduates to engage in career exploration and career construction.

## Introduction

In China, the expansion of college enrollment has brought about a sharp rise in the number of college graduates in the past 30 years, leading to more and more fierce competition for college students’ employment (Barr and Turner, 2013; Jackson, 2021), and the problem of “employment difficulties” of college students has been increasingly exposed and focused by the public and researchers (Ye et al., 2016; Chen et al., 2022). University graduation is a critical stage for college students to change their career roles and starting watershed in their career development (Wu et al., 2016); Although taking postgraduate exam and studying abroad are welcomed by fresh graduates, the challenge of constructing a career through job hunting and interviewing at graduation is still the most direct choice for most people (Donald et al., 2018). However, in the dual context of the COVID-19 and graduation season, the tense economy status and fierce competition for jobs caused by the shutdown of enterprises due to the impact of the epidemic; simultaneously, the job characteristics of college students with little work experience, low job proficiency, and long training cycles are two major causes of uncertain career development (Schwartz, 2016; Song and Zhou, 2020).

Career exploration is a prerequisite for career planning and a key part of career development for college students (Super, 1957). After career exploration, individuals plan their future careers based on the information obtained from career exploration (Super, 1980) and try to adapt their careers (Koen et al., 2012), influencing their future career development (Bartley and Robitschek, 2000; Ling et al., 2022). Self-regulation theory holds that the clearer an individual’s self-knowledge is, the easier it is for the self-regulation system to drive an individual to make efforts for his or her own goals and then engage in a series of actions (Bandura, 1991; Karoly, 1993). College students’ hopes and aspirations for future jobs will drive them to engage in job-hunting behaviors (Kanfer et al., 2001). College students with future work self-salience will be willing to act for career development and conduct career exploration (Zhang et al., 2014; Taber and Blankemeyer, 2015). College students who have a clear future job will conduct career exploration to build their career and show more adaptability to career uncertainty (Guan et al., 2014; Taber and Blankemeyer, 2015).

Second, career adaptability is a psychological construct that individuals use to cope with current and future career tasks, which varies with environment interaction (Savickas and Porfeli, 2012). Social cognitive career theory suggests that the environment, individual differences, and behaviors interact will influence an individual’s career development (Lent et al., 2000). Environmental factors that college students perceive as teacher helping them with their career exploration, along with their own effective part-time behaviors, may interact with college students’ future work clarity, career exploration, and collectively influence their career and adaptability (Martin and Sinclair, 2007; Jaworski et al., 2018; Pan et al., 2018). Due to the policy impact of college enrollment expansion (Goldrick-Rab, 2010) and the economic downturn caused by the COVID-19 epidemic, there are challenges for college students to develop and construct their careers (Mok et al., 2021). In addition, whether the two boundary conditions of teacher support and effective part-time behavior of college students positively affect the career exploration and future career adaptability of college students with future job clarity, which has a guiding role in the construction of college students’ future careers, and enhancing the employment rate and adaptability of college students, as well as the expansion value of career education in colleges and universities.

**FIGURE 1:**
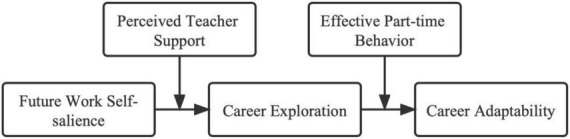
Preliminary model diagram of the study.

Thus, based on self-regulation theory to explore the mediating role of career exploration in future work self- salience and career adaptability, and on social cognitive career theory to explore the moderating effect of teacher support and effective part-time behavior in this indirect role, we can better explain the influence mechanism of career adaptability, and have more theoretical and practical significance. In view of this, this study addresses the shortcomings of previous studies and tries to solve the problem of “employment difficulties” for college students in mainland China, as well as to contribute to helping college students to have clearer career development under the economic downturn caused by the normalization of the epidemic, and to provide theoretical and practical suggestions for colleges and enterprises to carry out career guidance work. The research model is shown in Figure 1.

## Theory and hypothesis

### The relationship between future work self-salience and career adaptability

Career adaptability is a social psychological construction (Savickas and Porfeli, 2012) that is a core competency for individuals to achieve long-term career success (Hirschi, 2009) and exerts a positive predictive effect on future employment status (Koen et al., 2010; Guan et al., 2014), employment quality (Guan et al., 2013), career development (Ebberwein et al., 2004), and job promotion (Tolentino et al., 2013).Therefore, clarifying the formation mechanism of career adaptability of university students is beneficial for their future career development.

Strauss et al. (2012) first introduced the concept of future work self-salience, that is, the degree to which individuals can easily imagine their future job self-salience. Most subsequent studies have found that clarity and ease of imagining future work can help individuals better set career goals and clarify the behavioral paths they need to take (Taber and Blankemeyer, 2015; Arif et al., 2017). According to self-regulation theory: the clearer an individual is about himself, the easier it is for his self-regulation system to drive him toward a goal and thus show more practical actions (Karoly, 1993). College students with clear future work self-salience are more likely to motivate themselves to set goals for their future career development, exploring and making attempts to their career goals and showing higher career adaptability (Strobel et al., 2013). Second, college students with future work self-salience will actively seek feedback and proactively adjust their behaviors for career development to accommodate work and job search behaviors (Zhang et al., 2014). Furthermore, college students who are clear about their future job will be less bound by their environment, clarify their career goals, identify opportunities, and adopt a range of behaviors to accommodate their career development (Bateman and Crant, 1993). Finally, college students who are clear about their future jobs will actively engage in a variety of career planning, obtain career support, work to overcome obstacles, and demonstrate higher levels of career resilience (Seibert et al., 1999; Guan et al., 2014; Joanne Chan and Chan, 2021). Therefore, hypothesis 1 is proposed:

***Hypothesis 2: Future work self-salience positively influences career adaptability.***

### The mediating role of career exploration

In career, career exploration not only helps students move from school to the workplace, but also contributes to future job transitions (Flum and Blustein, 2000). Career exploration is the act of individuals gathering and analyzing information about their characteristics and job aspects (Stumpf et al., 1983). The act of career exploration positively affects short- and long-term career outcomes, such as better career decisions (Feldman and Bolino, 1996), more interview chances (Cheung and Jin, 2016), higher pay, and higher job satisfaction and happiness (Maggiori et al., 2013). During the course of career development, individuals will use career exploration to gain information and make decision planning for their future career, which influences their future career development (Werbel, 2000). An individual who is clear about their future job will actively gather information, engage in career exploration activities, and make more proactive career adaptive behaviors (Kaminsky and Behrend, 2015).

Career exploration is a self-regulatory process (Kanfer et al., 2001). Self-regulation theory suggests that individuals will self-regulate by dividing themselves into: self-observation, self-judgment, and self-response (Bandura, 1991). Future work self-salience represents an individual’s observation and judgment of future jobs (Strobel et al., 2013). Based on the information observed and judged, with clear career goals, the behavioral response of career exploration will be carried out (Côté et al., 2006). In addition, future work self-salience is a willingness of the individual and serves as an important prerequisite for goal generation (Parker et al., 2010). College students who are clear about the goals they hope to achieve will drive how they look for jobs, how they adapt to the future, and how they deal with the various issues that arise in their careers. Therefore, college students with future work self-salience can fully appreciate their expectations and ambitions for their future jobs, thus forming a clear job search goal and engaging in job search behavior by conducting career exploration for this goal (Zhao et al., 2022). At the same time, after completing career exploration with clear goals, college students will be more adaptable to tasks, problems, and changes in their future jobs to cope with career changes and career development to accomplish their career goals. Therefore, hypothesis 2 is put forward:

***Hypothesis 2: Future work self-salience indirectly influences career adaptability through career exploration.***

### The moderating role of perceived teacher support

Social cognitive career theory holds that individuals do not live in a vacuum and that the choices they make are influenced by factors in themselves and their surroundings (Lent et al., 2000, 2002). In particular, when individuals feel positively supported by their environment, they will act positively to achieve their goals (Lent et al., 2001). School and teachers play the most important influence on career development when college students transition from a student status in school to a professional status (Bronfenbrenner and Evans, 2000). Teacher support contributes to students’ confidence, attitudes, and behaviors (van der Ross et al., 2022). Perceived teacher support refers to students’ perceived support from teachers for their learning, attitudes, and abilities (Babad, 1990). Teacher support serves as an external stimulus for college students as they engage in career exploration, providing adequate support and encouragement and reducing deviant behavior (Wang and Eccles, 2012).

College students with future work self-salience are clear about their job hunting goals and know when they are going to look for a job of any type. Individuals who set goals for their future jobs will be motivated to make efforts to achieve their goals and engage in career exploration; At this time, if teachers give college students competence affirmation and career guidance support, college students will be motivated to engage in specific exploration behaviors in order to achieve their goals (Bandura, 1991). Next, according to social cognitive career theory-individual career development is influenced by both individual psychology and social environment (Lent et al., 2000). Based on environmental support from teachers, and their own unwavering job hunting goals and clear future jobs, college students will actively submit resumes, participate in interviews with an optimistic attitude, and engage in positive and effective career exploration behaviors (Lee et al., 2016). Moreover, good support from teachers at school can effectively facilitate the satisfaction of individual psychological needs, create a healthy psychological state, and alleviate the anxiety of uncertainty (Forster et al., 2020), promoting college students to change from the psychological level of clear future work to the behavioral level of career exploration with positive teacher support. Finally, in order to further expand the boundary conditions of the influence effect of future job self-salience among college students, efforts are made to explore the mechanism of the influence of the contextual variable of teacher support on the role of future work self-salience and career exploration, and Hypothesis 3 is proposed:

***Hypothesis 3: Teacher support positively moderates the positive relationship between future work self-salience and career exploration, and the stronger the perceived teacher support is, the stronger the positive relationship between future work self-clarity and career exploration is. Conversely, the relationship is weaker.***

### The moderating role of effective part-time job behavior

Individual differences, environmental factors, and one’s own behavior affect individual career development through interactions that positively influence career interests, career values, perceptions of career success, and career resilience according to social cognitive career theory (Lent and Brown, 1996). Part-time behavior among college students is an effective approach to know about jobs, clarify future work, and improve work adaptability before formally entering society (Helyer and Lee, 2014; Brooks and Youngson, 2016). Effective part-time behavior among college students is a value-conditioned resource related to future job development and career success (Jacoby, 2006). Such effective part-time work behavior facilitates the transition from career exploration to future job adaptability under the premise of clear career goals, promotes career identity, and enhances job search exploration, thus acquiring job adaptability (Yizhong and Hailing, 2016).

Career exploration, as one of the most critical aspects of career development, can effectively enhance college students’ future work adaptability (Super, 1957). Through career exploration, college students satisfy their self-efficacy for work (Werbel, 2000) and are more likely to show positive psychological capital, identify with their future job, and demonstrate high levels of resilience (Porfeli and Savickas, 2012). Still, according to the social cognitive career theory, self-differentiated behaviors affect career development, and effective part-time behaviors help optimize the positive job resilience generated by career exploration. College students can enhance their professionalism and teamwork through part-time behavior, and the qualities generated by such part-time can help facilitate career exploration for future job adaptation. Meanwhile, in order to explore the boundary role of effective part-time behavior on career exploration and career adaptability and to compensate for the differential contextual effects of their own part-time behavior in the process of college students’ career development, Hypothesis 4 is proposed:

***Hypothesis 4: College students’ effective part-time job behavior positively moderates the positive relationship between career exploration and career adaptation. The stronger the degree of effective part-time job behavior is, the stronger the positive relationship between career exploration and career adaptation is. Conversely, the relationship is weaker.***

### Mediation model with moderation

Integrating Hypothesis 2, Hypothesis 3 and Hypothesis 4, this study proposes a mediated model with moderation. When college students perceive the support given by teachers: individuals who have future work self-salience will fully experience their hopes and imagination of their future work, and thus set clear and explicit goals for career exploration according to their actual situation; At the same time, when college students have also engaged in part-time behavior related to their future work, and such part-time work is effective and developmental, college students will transfer the professional qualities and resources acquired in part-time to the jobs they have explored and show more adaptive behaviors. On the contrary, if college students do not receive career guidance and support from teachers during career exploration and career construction, and do not participate in part-time behavior that are beneficial to future job development, even if they have clear knowledge of future jobs, it is difficult to implement career exploration and adapt to future jobs, which affects career development. In view of this, hypothesis 5 is proposed:

***Hypothesis 5: Teacher support and college students’ effective part-time behavior positively moderate the indirect effect of future work self-salience on career adaptability through career exploration. The stronger the degree of teacher support and effective part-time behavior is, the stronger the indirect effect is. On the contrary, this indirect effect is weaker.***

## Research methods

### Research subjects and collection procedures

This study selects college students who are in the career selection period as the research object. According to the classification of career stages, it is generally accepted that young people around the age of 20 are in the exploratory stage, during which young people gradually develop career-related cognition (Super, 1953). In addition, the school-to-workplace transition process affects individuals’ future career development and career outcomes, and such age group is a critical period for developing clear career directions and setting career goals (Dietrich et al., 2012).

The specific study sample was selected from college students in Shanghai, Chongqing, Guangdong Province, and Jiangsu Province, which could meet the requirement of sample diversity because the student was from all over the country. To reduce the effect of common method bias (CMB), this study used a multi-stage completion approach for questionnaire collection as suggested by Podsakoff et al. (2003). Data were collected at the first time point for future work self-salience, perceived teacher support, and control variables with data collected at the second time point about career exploration and effective part-time behavior and at the third time point about career adaptability. Each time interval was one month with entire survey lasting three months (February to May 2022). Furthermore, the survey was adopted anonymously in order to avoid concerns of the respondents about the questionnaire items. The respondents were also informed before the official survey that their personal information and the content of the survey would be kept confidential and used only for this academic study.

At the first time point, a total of 920 questionnaires were distributed and 900 were collected with loss rate of 2.2%. At the second time point, a total of 900 questionnaires were sent out and 885 were recovered with loss rate of 1.7%. At the third time point, 885 questionnaires were issued and 855 were received with loss rate of 3.9%. At the end of the whole survey, a total of 840 valid questionnaires were obtained by taking student ID as the basis for three times matching and excluding the missed and wrongly filled questionnaires. The effective rate of this questionnaire was 91.3%. According to the survey results, descriptive statistical analysis was conducted and the following results were as follows: for the overall sample, there were 375 male students and 465 female students; 44 freshmen, 213 sophomores, 151 juniors and 432 seniors; The average age was 19.43 years old.

### Measuring tools

This study draws on established scales to ensure the reliability and validity of the questionnaire. Before investigation and research, the English scales were accurately translated into Chinese according to the standard translation and back-translation procedure (Brislin, 1986) and was repeatedly checked with the questionnaire distribution team. A 5-point Likert scale (1 to 5 in the questionnaire indicates “strongly disagree” to “strongly agree”) was used throughout the study.

Future work self-salience: Future work self-salience scale developed by Strauss et al. (2012) was adopted. The questionnaire has a total of 5 questions. Examples of questions are: “I can easily imagine my future job” and “I am clear about who and what I want to be in my future job.” The scale has an alpha coefficient of 0.912 in this study.

Teacher support: A questionnaire was applied to measure college students’ perception of teacher support behavior, with 19 items in total according to the scale compiled by Ou (2005). Examples of questions are “My teacher is always gentle with me” and “My teacher often encourages me in my study and life.” This scale was used in the study and the Conbrach’s alpha coefficient is 0.939.

Career exploration: The Career Exploration Scale developed by Stumpf et al. (1983) was employed to measure students’ career exploration activities with a total of 17 items in this questionnaire. Examples of questions are: “I know a lot of information about the career field I have focused on” and “I will try different career activities.” The scale has a Conbrach’s alpha coefficient of 0.913 in this study.

Effective part-time behavior: There are 4 questions with reference to Mingqian and Sanman (2021) part-time behavior scale. Examples of questions are “Part-time jobs are a boon to employment” and “In general, the part-time job I choose is more related to my major.” This scale has an alpha coefficient of 0.901 in this study.

Career Adaptability: The Career Adaptability Scale, as revised by Hou et al. (2012), was used to measure students’ adaptability to their future careers. The questionnaire has 24 questions, covering four dimensions of career concern, control, curiosity, and self-confidence, and Examples of questions are: “I can think about what my future will be like” and “I will make my own decisions.” The scale has a Conbrach’s alpha coefficient of 0.948 in this study.

Control variables: Based on previous studies, gender, age, and education have been found to influence individual career adaptability (Guan et al., 2013; Cai et al., 2015). In addition, students’ place of origin also has an impact on career development and job resilience (Garriott, 2020; Kim and Smith, 2021). To more accurately validate the model, gender, age, grade, and birthplace were measured as control variables in this study.

### Data analysis methods

This study used SPSS 21.0 for Harman’s one-way test, descriptive statistics, correlation analysis, and multiple regression analysis, and Amos 21.0 for confirmatory factor analysis. In testing for mediating effects, this study used the three-step method of Baron and Kenny (1986) and combined it with the Bootstrap technique (using the PROCESS program) (Hayes, 2017) to estimate confidence intervals for mediating effects. In testing for mediators with moderation, this study relied on Edwards and Lambert’s (2007) study and integrated with the bootstrap technique (Bootstrap) to test for the significance of the values and differences of indirect effects under high and low moderating variables.

## Research results

### Common method deviation test

In the research investigation, the multi-stage fill-in approach suggested by Podsakoff et al. (2003) is followed to control for possible common method bias in the study at the methodological level (Podsakoff et al., 2003). At the data level, Harman’s one-way test was performed on the data collected and the percentage of explained variance by the first factor was found to be 27.79%, a rate that is less than the 40% criterion (Podsakoff et al., 2003). Also, as can be seen in Table 1, the fitting results of the confirmatory factor analysis of the one-factor model were also poor (χ2 = 18760.698, df = 2225, RMSEA = 0.094, SRMR = 0.144, CFI = 0.705, TLI = 0.689), indicating that there was no serious common method bias among the variables.

**TABLE 1:** Results of confirmatory factor analysis (*N* = 840).

Model	χ 2	df	Δχ 2	RMSEA	SRMR	CFI	TLI
Five-factor model (hypothesis)	7322.779	2215		0.050	0.047	0.909	0.904
Four-factor model (A + B)	9187.303	2219	1864.524***	0.061	0.062	0.876	0.869
Four-factor model (C + D)	9823.666	2219	2500.887***	0.064	0.094	0.865	0.857
Three-factor model (A + B + C)	13889.446	2222	6566.667***	0.079	0.174	0.792	0.781
Three-factor model (B + C + D)	13070.504	2222	5747.725***	0.076	0.189	0.807	0.796
Two-factor model (A + B + C + D)	14857.165	2224	7534.386***	0.082	0.187	0.775	0.763
One-factor model (A + B + C + D + E)	18760.698	2225	11437.919***	0.094	0.144	0.705	0.689

A: Future work self-salience; B: Perceived teacher support; C: Career exploration; D: Effective part-time behavior; E: Career adaptability; + indicating integration.

***p < 0.001, **p < 0.01, *p < 0.05.

### Confirmatory factor analysis

In this study, the following fitting indicators were selected to judge the model fit, including the chi-square difference must reach a significant level, the root mean squared error of approximation (RMSEA) must be less than 0.08, and the comparative fitness index (CFI) and Tucker-Lewis index (TLI) must be greater than 0.9. A series of competing models were compared in this study, and the results of the analysis are shown in Table 1. It can be seen from Table 1 that the model fit of the five-factor model (χ2 = 7322.779, df = 2215, RMSEA = 0.050, SRMR = 0.047, CFI = 0.909, TLI = 0.904) was better than the other competing models in this study. Furthermore, all the fitness indicators of the five-factor model passed the test. Accordingly, all the variables in this study were distinguishable.

### Correlation analysis

The results of the correlation analysis between control variables and variables are shown in Table 2. From Table 2, it can be known that there is a significant positive correlation between all variables, providing a preliminary basis for hypothesis testing of the model. There is a significant positive relationship between future work self-salience and career adaptability (*r* = 0.364, *p* < 0.001), which can initially prove the validity of hypothesis 1.

**TABLE 2:** Mean values, standard deviations and correlation coefficients of variables (*N* = 840).

Variables	Mean values	Standard deviations	1	2	3	4	5	6	7	8
1 Gender	0.550	0.497								
2 Age	19.433	1.342	0.012							
3 Grade	15.156	0.977	–0.070*	–0.812***						
4 Place of origin	0.924	0.266	–0.177***	0.173***	–0.198***					
5 Future work self-salience	3.545	0.662	–0.207***	0.023	0.010	0.063				
6 Perceived teacher support	3.706	0.569	–0.194***	–0.020	0.020	0.113**	0.495***			
7 Career exploration	3.654	0.531	–0.204***	0.061	–0.051	0.103**	0.356***	0.361***		
8 Effective part-time behavior	4.079	0.658	–0.164***	–0.027	0.011	0.130***	0.375***	0.703***	0.281***	
9 Career adaptability	4.133	0.513	-0.153***	0.049	–0.035	0.103**	0.364***	0.356***	0.778***	0.259***

***p < 0.001, **p < 0.01, * *p* < 0.05.

### Hypothesis testing results

1. Test results of main effect

As shown by Model 3 in Table 3, future work self-salience presents a significant positive relationship with career adaptability (β = 0.35, *p* < 0.001). Hypothesis 1 is supported.

1. Test results of mediating effect

**TABLE 3:** Hypothesis testing model.

Variables	Career exploration		Career adaptability			
	Model 1	Model 2	Model 3	Model 4	Model 5	Model 6
Control variable						
Gender	–0.13***	–0.11**	–0.07*	0.03	0.02	0.03
Age	0.01	0.02	0.02	0.01	0.03	0.02
Grade	–0.05	–0.04	–0.02	0.02	0.03	0.02
Place of origin	0.05	0.03	0.06	0.03	0.03	0.03
**Independent variable**						
Future work self-salience	0.33***	0.20***	0.35***	0.10***		0.07*
**Mediating variable**						
Career exploration				0.74***	0.76***	0.07***
**Moderating variable**						
Perceived teacher support		0.22***				0.07*
Effective part-time behavior					0.04	0.02
**Interaction items**						
Future work self-salience *Perceived teacher support		0.10**				0.02*
Career exploration*Effective part-time behavior					0.08***	0.06*
R^2^	0.15	0.20	0.14	0.61	0.61	0.62
F	29.60***	29.66***	28.17***	222.91***	189.39***	136.27***

***p < 0.001, **p < 0.01, * *p* < 0.05.

According to Model 4 in Table 3, there is a significant positive relationship between future work self-salience and career adaptability (β = 0.10, *P* < 0.001), and a significant positive relationship between career exploration and career adaptability (β = 0.74, *P* < 0.001), verifying the indirect effect of future work self-salience on career adaptability through career exploration. To clarify this indirect effect again, this study uses the Bootstrap method test (Hayes, 2017). The Bootstrap method test for the mediating effect is shown in Table 4, where both the direct and indirect effects of future work self-salience and career adaptability do not include zero at the 95% confidence interval. Thus, it can be confirmed that career exploration plays a partially mediating role in the relationship between future work self-salience and career adaptability. Hypothesis 2 is supported.

1. Test results of moderating effects

**TABLE 4:** Bootstrap test for mediating effects.

Mediating effect	Effect value	Standard error	95% of confidence interval	
			Lower confidence limit	Upper confidence limit
Indirect effect	0.19	0.02	0.04	0.11
Direct effect	0.08	0.02	0.15	0.23

Bootstrap sample size *N* = 5000.

**FIGURE 2:**
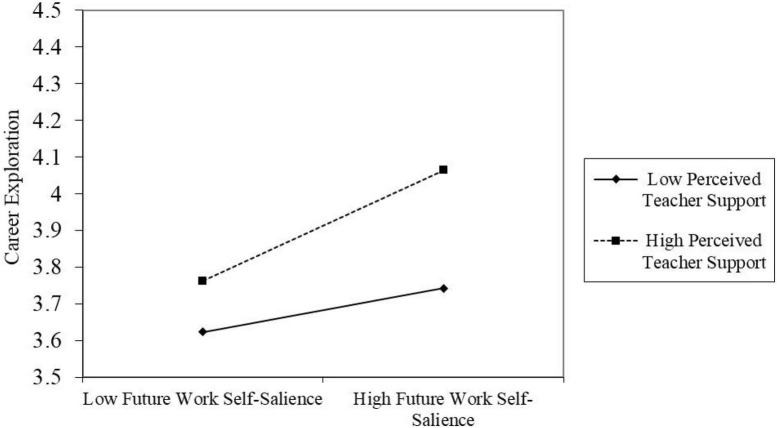
The moderating role of perceived teacher support between future work self-salience and career exploration.

Confirming the moderating effect of perceived teacher support. As displayed by Model 3 in Table 3, the interaction item between future work self-salience and perceived teacher support presents a prominent positive relationship with career exploration (β = 0.10, *p* < 0.01). Simultaneously, Bootstrap method test of moderating effect is shown in Table 5. At 95% confidence interval, with low level of teacher support, the indirect effect of future work self-salience on career exploration is low (with effect value of 0.09), while with high level of teacher support, the indirect effect of future work self-salience on career exploration is high (with effect value of 0.23). This research is determined by using Aiken et al. (1991) to regulate the high and low levels of the moderating variables in an attempt to clarify such moderating effect. As seen in Figure 2, the positive relationship between future work self-salience and career exploration is stronger when the degree of teacher support is higher. Hypothesis 3 is supported.

**TABLE 5:** Bootstrap test of the moderating effect with perceived teacher support.

Moderating effect	Effect value	Standard error	95% of confidence interval	
			Lower confidence limit	Upper confidence limit
Low (–1SD)	0.09	0.04	0.01	0.17
Medium	0.16	0.03	0.10	0.22
High (+ 1SD)	0.23	0.03	0.16	0.29

Bootstrap sample size *N* = 5000.

**FIGURE 3:**
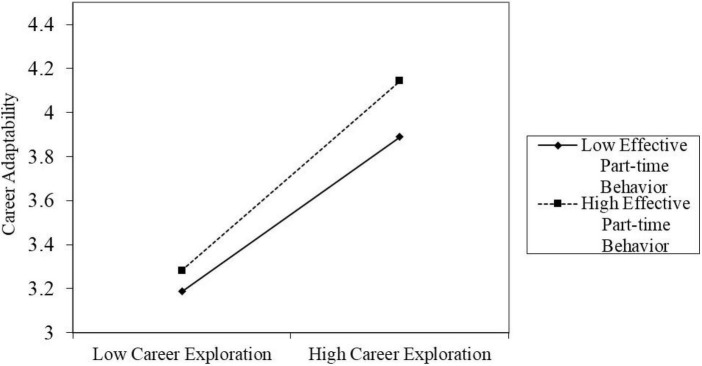
The moderating role of effective part-time behavior between career exploration and career adaptability.

Confirming the moderating effect of effective part-time behavior. As shown in Model 5 of Table 3, the interaction item between career exploration and effective part-time behavior takes on a remarkable positive relationship with career adaptability (β = 0.08, *p* < 0.001). Meanwhile, the Bootstrap method test for the moderating effect is presented in Table 6, the indirect effect of career exploration on career adaptability is weaker at low levels of effective part-time behavior (with effect value of 0.66) and stronger at high levels of effective part-time behavior (with effect value of 0.81). This research is determined by using Aiken et al. (1991) to regulate the high and low levels of the moderating variables to clarify such moderating effect. As seen in Figure 3, the positive relationship between career exploration and career adaptability is stronger at higher levels of effective part-time behavior. Hypothesis 4 is supported.

1. Test results of mediating effects with moderation

**TABLE 6:** Bootstrap test of the moderating effect of effective part-time behavior.

Moderating effect	Effect value	Standard error	95% of confidence interval	
			Lower confidence limit	Upper confidence limit
Low (–1SD)	0.66	0.03	0.60	0.72
Me–dium	0.73	0.02	0.69	0.78
High (+ 1SD)	0.81	0.03	0.76	0.87

Bootstrap sample size *N* = 5000.

This study employs the effect values of indirect effects of Bootstrap’s method test at high and low levels of moderating variables (Edwards and Lambert, 2007) in a bid to verify whether perceived teacher support and effective part-time behavior moderated the indirect effect of future work self-salience on career adaptability through career exploration. As understood in Table 7, under the condition of high degree of teacher support and high level of effective part-time job behavior, the indirect effect of future work self-salience on career adaptability through career exploration is 0.18, whose value is [0.13,0.23] at 95% confidence interval. With low levels of teacher support and effective part-time behavior, the indirect effect of future work self-salience through career exploration on career adaptability is 0.06, with value of [0.01,0.11] at 95% confidence interval. At the same time, given the inconsistency between high and low levels of teacher support and effective part-time behavior, the indirect effects of future work self-salience on career adaptability through career exploration are 0.07 and 0.15, respectively, which also remained significant at the 95% confidence interval. In conclusion, the higher the degree of perceived teacher support and effective part-time job behavior, the stronger the indirect effect of future work self-salience on career adaptability through career exploration. Hypothesis 5 is supported.

**TABLE 7:** Bootstrap test for mediating effects with moderation.

Independent variable	Moderating variable (perceived teacher support)	Moderating variable(effective part-time behavior)	Indirect effect	Standard error	95% of confidence interval	
					Lower confidence limit	Upper confidence limit
Perceived teacher support	low(–SD)	low(–SD)	0.06	0.03	0.01	0.11
	low(–SD)	high(+ SD)	0.07	0.03	0.01	0.18
	high(+ SD)	low(–SD)	0.15	0.02	0.11	0.20
	high(+ SD)	high(+ SD)	0.18	0.03	0.13	0.23

Bootstrap sample size *N* = 5000.

## Conclusion

Career adaptability is significant for college graduates to conduct career construction (Rudolph et al., 2017). Starting from self-regulation theory and social cognitive career theory, this study delves into the mechanism inherent in future work self-salience through career exploration on career adaptability and discusses the moderating role of teacher support, effective part-time behavior in this mechanism. It attempts to propose theoretical and practical guidance for college students on this issue of career construction.

## Discussion

### Theoretical contributions

First, this study systematically identifies the causes of career adaptability. A moderated mediation model is proposed in terms of individual psychology, environmental support and individual’s own behavior. The theoretical basis of career adaptability is extended by exploring how college students can acquire career adaptability.

Second, this study proposes a positive relationship between future work self-salience and career adaptability, enriching the exploration of career adaptability in terms of psychological factors. Meanwhile, it is consistent with the predictions of Career Construction Theory (CCT) and The Model of Proactive Motivation (Guan et al., 2014).

Third, the mediating role of career exploration. College students’ future work self-salience (psychological level) through career exploration (behavioral level) affects future work adaptability. The bridging role of career exploration is systematically shown; At the same time, this finding not only supports the self-regulation theory, but also makes some supplementary studies on career adaptability.

Fourth, two moderating variables like individual perceived teacher support and effective part-time behavior are introduced. The indirect effect of future work self-salience on career adaptability through career exploration is stronger when college students feel more supported by teachers or engage in more effective part-time jobs. This finding exactly supports social cognitive career theory (Lent and Brown, 1996). It also provides some reference for follow-up research on teachers’ occupation and part-time behavior of college student.

### Practical contributions

From the perspective of career construction of college students and college career guidance, this study provides some practical suggestions for the management of the topic of “how college students can be more comfortable with their careers”.

#### Improving career exploration behavior and career adaptability

This study takes college students as the research object and explores their career exploration behavior and career adaptability from the psychological factors. Based on the findings of the study, the following suggestions are made. Primarily, college students need to increase their own clarity about their future jobs to cope with employment problems. In the next place, college students should clarify their career goals as early as possible, which is conducive to more effective job exploration and job adaptation. Thirdly, they should prepare for their career by clarifying what kind of job they want to do, when they want to do, and how to do.

#### Situation role of teacher support and part-time behavior

This study considers the contextual role of teacher support and individual part-time behavior based on the social cognitive career theory. When providing career guidance to college students, colleges should make students perceive support from schools and teachers, which can guide them to actively engage in career construction. Meanwhile, students should actively realize that effective part-time jobs are beneficial to their future work growth and actively engage in part-time jobs and internships. Premised on the above research findings, the following suggestions are made. First, colleges and universities should establish professional career guidance teachers, offer professional career guidance courses and strengthen the efforts to provide college students with career guidance (Solberg et al., 2002; Magee et al., 2022). Second, increase the proportion of social practice courses in the curriculum design, and guide college students to participate in practical activities that are beneficial to career development (Hrivnak, 2019). Third, provide employment opportunities appropriately (Yang et al., 2002). For example, carry out school-enterprise cooperation activities and strengthen the efforts of holding campus job fairs (Weiming et al., 2016).

#### Family support to help with career construction

In addition to school support, family support is also very crucial for college students’ careers, especially in Chinese society where career choice and job search go hand in hand with family (Hansen and Pang, 2008). As parents, friends and relatives, they should not only give financial support to college students, but also give more networking support. During the period of career construction, more help should be given in time to successfully solve the employment problem and start career.

## Limitation and future research directions

Firstly, the sample data of this study are all obtained from self-reported questionnaires of college students, and it is suggested that multiple sources will be used to obtain survey data subsequently.

Secondly, the college students in this study are all undergraduate students. Whether the career construction or work adaptability of postgraduate and doctoral students is universal is still worthy of follow-up research. Therefore, it is suggested that future studies should take students at different academic levels as research subjects to extend the applicability of the study.

Thirdly, this study takes into account the influence of college students’ psychological factors, college environment and teachers’ support on career adaptability. However, the influence of family atmosphere and parental support on the construction of college students’ career is not considered. Therefore, it is suggested that future research can be conducted from a perspective of cross-level research of family, society and school.

Fourthly, the research method of this study adopts questionnaire survey, which is a single research method. It is suggested that the quasi-experimental method can be applied to invite college students to participate in relevant experiments to do following research on career adaptability.

## Data availability statement

The raw data supporting the conclusions of this article will be made available by the authors, without undue reservation.

## Author contributions

All authors listed have made a substantial, direct, and intellectual contribution to the work, and approved it for publication.

## References

[B1] Aiken L. S. West S. G. Reno R. R. (1991). *Multiple regression: Testing and interpreting interactions.* Thousand Oaks, CA: sage.

[B2] Arif M. Ilyas Sindhu M. Faiza Urooj S. Haider Hashmi S. (2017). Impact of abusive supervision on turnover intention through future work self-salience and organization-based self-esteem. *Int. J. Organ. Leadersh.* 6 481–490. 10.33844/ijol.2017.60260

[B3] Babad E. (1990). Measuring and changing teachers’ differential behavior as perceived by students and teachers. *J. Educ. Psychol.* 82 683–690. 10.1037/0022-0663.82.4.683

[B4] Bandura A. (1991). Social cognitive theory of self-regulation. *Organ. Behav. Hum. Decis. Process.* 50 248–287. 10.1016/0749-5978(91)90022-L

[B5] Baron R. M. Kenny D. A. (1986). The moderator–mediator variable distinction in social psychological research: Conceptual, strategic, and statistical considerations. *J. Pers. Soc. Psychol.* 51 1173–1182. 10.1037/0022-3514.51.6.1173 3806354

[B6] Barr A. Turner S. E. (2013). Expanding enrollments and contracting state budgets: The effect of the great recession on higher education. *Ann. Am. Acad. Polit. Soc. Sci.* 650 168–193. 10.1177/0002716213500035

[B7] Bartley D. F. Robitschek C. (2000). Career exploration: A multivariate analysis of predictors. *J. Vocat. Behav.* 56 63–81. 10.1006/jvbe.1999.1708

[B8] Bateman T. S. Crant J. M. (1993). The proactive component of organizational behavior: A measure and correlates. *J. Organ. Behav.* 14 103–118. 10.1002/job.4030140202

[B9] Brislin R. W. (1986). “The wording and translation of research instruments,” in *Field methods in cross-cultural research*, eds Lonner W. J. Berry J. W. (Thousand Oaks, CA: Sage Publications, Inc.), 137–164.

[B10] Bronfenbrenner U. Evans G. W. (2000). Developmental science in the 21st century: Emerging questions, theoretical models, research designs and empirical findings. *Soc. Dev.* 9 115–125. 10.1111/1467-9507.00114

[B11] Brooks R. Youngson P. L. (2016). Undergraduate work placements: An analysis of the effects on career progression. *Stud. High. Educ.* 41 1563–1578. 10.1080/03075079.2014.988702

[B12] Cai Z. Guan Y. Li H. Shi W. Guo K. Liu Y. (2015). Self-esteem and proactive personality as predictors of future work self and career adaptability: An examination of mediating and moderating processes. *J. Vocat. Behav.* 86 86–94. 10.1016/j.jvb.2014.10.004

[B13] Chen H. Liu F. Wen Y. (2022). The influence of college students’ core self-evaluation on job search outcomes: Chain mediating effect of career exploration and career adaptability. *Curr. Psychol.* 1–12. 10.1007/s12144-022-02923-4 [Epub ahead of print]. 35228786 PMC8865730

[B14] Cheung R. Jin Q. (2016). Impact of a career exploration course on career decision making, adaptability, and relational support in Hong Kong. *J. Career Assess.* 24 481–496. 10.1177/1069072715599390

[B15] Côté S. Saks A. M. Zikic J. (2006). Trait affect and job search outcomes. *J. Vocat. Behav.* 68 233–252. 10.1016/j.jvb.2005.08.001

[B16] Dietrich J. Parker P. Salmela-Aro K. (2012). Phase-adequate engagement at the post-school transition. *Dev. Psychol.* 48 1575–1593. 10.1037/a0030188 23127301

[B17] Donald W. E. Ashleigh M. J. Baruch Y. (2018). Students’ perceptions of education and employability: Facilitating career transition from higher education into the labor market. *Career Dev. Int.* 23 513–540. 10.1108/CDI-09-2017-0171

[B18] Ebberwein C. A. Krieshok T. S. Ulven J. C. Prosser E. C. (2004). Voices in transition: Lessons on career adaptability. *Career Dev. Q.* 52 292–308. 10.1002/j.2161-0045.2004.tb00947.x

[B19] Edwards J. R. Lambert L. S. (2007). Methods for integrating moderation and mediation: A general analytical framework using moderated path analysis. *Psychol. Methods* 12 1–22. 10.1037/1082-989X.12.1.1 17402809

[B20] Feldman D. C. Bolino M. C. (1996). Careers within careers: Reconceptualizing the nature of career anchors and their consequences. *Hum. Resour. Manage. Rev.* 6 89–112. 10.1016/S1053-4822(96)90014-5

[B21] Flum H. Blustein D. L. (2000). Reinvigorating the study of vocational exploration: A framework for research. *J. Vocat. Behav.* 56 380–404. 10.1006/jvbe.2000.1721

[B22] Forster M. Grigsby T. J. Gower A. L. Mehus C. J. McMorris B. J. (2020). The role of social support in the association between childhood adversity and adolescent self-injury and suicide: Findings from a statewide sample of high school students. *J. Youth Adolesc.* 49 1195–1208. 10.1007/s10964-020-01235-9 32297174

[B23] Garriott P. O. (2020). A critical cultural wealth model of first-generation and economically marginalized college students’ academic and career development. *J. Career Dev.* 47 80–95. 10.1177/0894845319826266

[B24] Goldrick-Rab S. (2010). Challenges and opportunities for improving community college student success. *Rev. Educ. Res.* 80 437–469. 10.3102/0034654310370163

[B25] Guan Y. Deng H. Sun J. Wang Y. Cai Z. Ye L. (2013). Career adaptability, job search self-efficacy and outcomes: A three-wave investigation among Chinese university graduates. *J. Vocat. Behav.* 83 561–570. 10.1016/j.jvb.2013.09.003

[B26] Guan Y. Guo Y. Bond M. H. Cai Z. Zhou X. Xu J. (2014). New job market entrants’ future work self, career adaptability and job search outcomes: Examining mediating and moderating models. *J. Vocat. Behav.* 85 136–145. 10.1016/j.jvb.2014.05.003

[B27] Hansen M. H. Pang C. (2008). Me and my family: Perceptions of individual and collective among young rural Chinese. *Eur. J. East Asian Stud.* 7 75–99. 10.1163/156805808X333929

[B28] Hayes A. F. (2017). *Introduction to mediation, moderation, and conditional process analysis: A regression-based approach.* NewYork, NY: Guilford publications.

[B29] Helyer R. Lee D. (2014). The role of work experience in the future employability of higher education graduates. *High. Educ. Q.* 68 348–372. 10.1111/hequ.12055

[B30] Hirschi A. (2009). Career adaptability development in adolescence: Multiple predictors and effect on sense of power and life satisfaction. *J. Vocat. Behav.* 74 145–155. 10.1016/j.jvb.2009.01.002

[B31] Hou Z. J. Leung S. A. Li X. Li X. Xu H. (2012). Career adapt-abilities scale—China form: Construction and initial validation. *J. Vocat. Behav.* 80 686–691. 10.1016/j.jvb.2012.01.006

[B32] Hrivnak G. A. (2019). The increasing importance of curriculum design and its implications for management educators. *J. Manage. Educ.* 43 271–280. 10.1177/1052562918822068

[B33] Jackson M. (2021). Expansion, enrollment, and inequality of educational opportunity. *Sociol. Methods Res.* 50 1215–1242. 10.1177/0049124119852376

[B34] Jacoby D. (2006). Effects of part-time faculty employment on community college graduation rates. *J. High. Educ.* 77 1081–1103. 10.1353/jhe.2006.0050 34409987

[B35] Jaworski C. Ravichandran S. Karpinski A. C. Singh S. (2018). The effects of training satisfaction, employee benefits, and incentives on part-time employees’ commitment. *Int. J. Hosp. Manage.* 74 1–12. 10.1016/j.ijhm.2018.02.011

[B36] Joanne Chan S. H. Chan K. T. (2021). The indirect effects of self-esteem and future work self on career adaptability factors: A study of Chinese undergraduate students. *J. Employ. Couns.* 58 50–73. 10.1002/joec.12157

[B37] Kaminsky S. E. Behrend T. S. (2015). Career choice and calling: Integrating calling and social cognitive career theory. *J. Career Assess.* 23 383–398. 10.1177/1069072714547167

[B38] Kanfer R. Wanberg C. R. Kantrowitz T. M. (2001). Job search and employment: A personality–motivational analysis and meta-analytic review. *J. Appl. Psychol.* 86 837–855. 10.1037/0021-9010.86.5.837 11596801

[B39] Karoly P. (1993). Mechanisms of self-regulaton: A systems view. *Ann. Rev. Psychol.* 44 23–52. 10.1146/annurev.ps.44.020193.000323

[B40] Kim J. Smith C. K. (2021). Traumatic experiences and female university students’ career adaptability. *Career Dev. Q.* 69 263–277. 10.1002/cdq.12272

[B41] Koen J. Klehe U. C. Van Vianen A. E. (2012). Training career adaptability to facilitate a successful school-to-work transition. *J. Vocat. Behav.* 81 395–408. 10.1016/j.jvb.2012.10.003

[B42] Koen J. Klehe U. C. Van Vianen A. E. Zikic J. Nauta A. (2010). Job-search strategies and reemployment quality: The impact of career adaptability. *J. Vocat. Behav.* 77 126–139. 10.1016/j.jvb.2010.02.004

[B43] Lee B. Porfeli E. J. Hirschi A. (2016). Between-and within-person level motivational precursors associated with career exploration. *J. Vocat. Behav.* 92 125–134. 10.1016/j.jvb.2015.11.009

[B44] Lent R. W. Brown S. D. (1996). Social cognitive approach to career development: An overview. *Career Dev. Q.* 44 310–321. 10.1002/j.2161-0045.1996.tb00448.x

[B45] Lent R. W. Brown S. D. Hackett G. (2000). Contextual supports and barriers to career choice: A social cognitive analysis. *J. Couns. Psychol.* 47 36–49. 10.1037/0022-0167.47.1.36

[B46] Lent R. W. Brown S. D. Hackett G. (2002). Social cognitive career theory. *Career Choice Dev.* 4 255–311.

[B47] Lent R. W. Brown S. D. Brenner B. Chopra S. B. Davis T. Talleyrand R. (2001). The role of contextual supports and barriers in the choice of math/science educational options: A test of social cognitive hypotheses. *J. Couns. Psychol.* 48 474–483. 10.1037/0022-0167.48.4.474

[B48] Ling H. Teng S. Liu X. Wu J. Gu X. (2022). Future work self salience and future time perspective as serial mediators between proactive personality and career adaptability. *Front. Psychol.* 13:824198. 10.3389/fpsyg.2022.824198 35572329 PMC9094421

[B49] Magee M. Kuijpers M. Runhaar P. (2022). How vocational education teachers and managers make sense of career guidance. *Br. J. Guidance Couns.* 50 273–289. 10.1080/03069885.2021.1948970

[B50] Maggiori C. Johnston C. S. Krings F. Massoudi K. Rossier J. (2013). The role of career adaptability and work conditions on general and professional well-being. *J. Vocat. Behav.* 83 437–449. 10.1016/j.jvb.2013.07.001

[B51] Martin J. E. Sinclair R. R. (2007). A typology of the part-time workforce: Differences on job attitudes and turnover. *J. Occup. Organ. Psychol.* 80 301–319. 10.1348/096317906X113833

[B52] Mingqian L. Sanman H. (2021). A study on the relationship between academic performance and perceived employability of college graduates. *High. Educ. Explor.* 110–116. 10.3969/j.issn.1673-9760.2021.09.017

[B53] Mok K. H. Xiong W. Ye H. (2021). COVID-19 crisis and challenges for graduate employment in Taiwan, Mainland China and East Asia: A critical review of skills preparing students for uncertain futures. *J. Educ. Work* 34 247–261. 10.1080/13639080.2021.1922620

[B54] Ou Y. D. (2005). *A research on the relation among teachers’ ex-pectation, self-conception of students’ academic achievement, students’ perception of teacher’s behavioral supporting and the study achievement.* Guilin: Guangxi Normal University. (In Chinese).

[B55] Pan J. Guan Y. Wu J. Han L. Zhu F. Fu X. (2018). The interplay of proactive personality and internship quality in Chinese university graduates’ job search success: The role of career adaptability. *J. Vocat. Behav.* 109 14–26. 10.1016/j.jvb.2018.09.003

[B56] Parker S. K. Bindl U. K. Strauss K. (2010). Making things happen: A model of proactive motivation. *J. Manage.* 36 827–856. 10.1177/0149206310363732

[B57] Podsakoff P. M. MacKenzie S. B. Lee J. Y. Podsakoff N. P. (2003). Common method biases in behavioral research: A critical review of the literature and recommended remedies. *J. Appl. Psychol.* 88 879–903. 10.1037/0021-9010.88.5.879 14516251

[B58] Porfeli E. J. Savickas M. L. (2012). Career adapt-abilities scale-USA form: Psychometric properties and relation to vocational identity. *J. Vocat. Behav.* 80 748–753. 10.1016/j.jvb.2012.01.009

[B59] Rudolph C. W. Lavigne K. N. Zacher H. (2017). Career adaptability: A meta-analysis of relationships with measures of adaptivity, adapting responses, and adaptation results. *J. Vocat. Behav.* 98 17–34. 10.1016/j.jvb.2016.09.002

[B60] Savickas M. L. Porfeli E. J. (2012). Career Adapt-Abilities Scale: Construction, reliability, and measurement equivalence across 13 countries. *J. Vocat. Behav.* 80 661–673. 10.1016/j.jvb.2012.01.011

[B61] Schwartz R. B. (2016). The career pathways movement: A promising strategy for increasing opportunity and mobility. *J. Soc. Issues* 72 740–759. 10.1111/josi.12192

[B62] Seibert S. E. Crant J. M. Kraimer M. L. (1999). Proactive personality and career success. *J. Appl. Psychol.* 84 416–427. 10.1037/0021-9010.84.3.416 10380421

[B63] Solberg V. S. Howard K. A. Blustein D. L. Close W. (2002). Career development in the schools: Connecting school-to-work-to-life. *Couns. Psychol.* 30 705–725. 10.1177/0011000002305003

[B64] Song L. Zhou Y. (2020). The COVID-19 pandemic and its impact on the global economy: What does it take to turn crisis into opportunity? *China World Econ.* 28 1–25. 10.1111/cwe.12349

[B65] Strauss K. Griffin M. A. Parker S. K. (2012). Future work selves: How salient hoped-for identities motivate proactive career behaviors. *J. Appl. Psychol.* 97 580–598. 10.1037/a0026423 22122111

[B66] Strobel M. Tumasjan A. Spörrle M. Welpe I. M. (2013). The future starts today, not tomorrow: How future focus promotes organizational citizenship behaviors. *Hum. Relat.* 66 829–856. 10.1177/0018726712470709

[B67] Stumpf S. A. Colarelli S. M. Hartman K. (1983). Development of the career exploration survey (CES). *J. Vocat. Behav.* 22 191–226. 10.1016/0001-8791(83)90028-3

[B68] Super D. E. (1953). A theory of vocational development. *Am. Psychol.* 8 185–190. 10.1037/h0056046

[B69] Super D. E. (1957). *The psychology of careers.* New York, NY: Harper & Row.

[B70] Super D. E. (1980). A life-span, life-space approach to career development. *J. Vocat. Behav.* 16 282–298. 10.1016/0001-8791(80)90056-1

[B71] Taber B. J. Blankemeyer M. (2015). Future work self and career adaptability in the prediction of proactive career behaviors. *J. Vocat. Behav.* 86 20–27. 10.1016/j.jvb.2014.10.005

[B72] Tolentino L. R. Garcia P. R. J. M. Restubog S. L. D. Bordia P. Tang R. L. (2013). Validation of the career adapt-abilities scale and an examination of a model of career adaptation in the philippine context. *J. Vocat. Behav.* 83 410–418. 10.1016/j.jvb.2013.06.013

[B73] van der Ross M. R. Olckers C. Schaap P. (2022). Engagement of academic staff amidst COVID-19: The role of perceived organisational support, burnout risk, and lack of reciprocity as psychological conditions. *Front. Psychol.* 13:874599. 10.3389/fpsyg.2022.874599 35602742 PMC9121175

[B74] Wang M. T. Eccles J. S. (2012). Social support matters: Longitudinal effects of social support on three dimensions of school engagement from middle to high school. *Child Dev.* 83 877–895. 10.1111/j.1467-8624.2012.01745.x 22506836

[B75] Weiming L. Chunyan L. Xiaohua D. (2016). Ten years of entrepreneurship education at chinese universities: Evolution, problems, and system building. *Chin. Educ. Soc.* 49 198–216. 10.1080/10611932.2016.1218250

[B76] Werbel J. D. (2000). Relationships among career exploration, job search intensity, and job search effectiveness in graduating college students. *J. Vocat. Behav.* 57 379–394. 10.1006/jvbe.2000.1746

[B77] Wu J. Dong Y. Xiong J. Cao Y. (2016). The mediating role of achievement motivation between college students’ proactive personality and career adaptability and its gender differences. *Psychol. Dev. Educ.* 32 547–556. (In Chinese).

[B78] Yang E. Wong S. C. Hwang M. H. Heppner M. J. (2002). Widening our global view: The development of career counseling services for international students. *J. Career Dev.* 28 203–213. 10.1177/089484530202800305

[B79] Ye B. Zheng Q. Liu L. Fang X. (2016). The effect of career exploration on job-searching behavior of college students: The mediating role of job searching self-efficacy and the moderation role of emotion regulation. *Psychol. Dev. Educ.* 32 691–697. (In Chinese).

[B80] Yizhong X. Hailing L. (2016). A longitudinal study of the effects of employability and job search behaviors on job search outcomes of college graduates. *Manage. Rev.* 28 109–120.

[B81] Zhang Y. Liao J. Yan Y. Guo Y. (2014). Newcomers’ future work selves, perceived supervisor support, and proactive socialization in Chinese organizations. *Soc. Behav. Pers. Int. J.* 42 1457–1472.

[B82] Zhao F. Li P. Chen S. Qin J. (2022). Career exploration and career decision self-efficacy in northwest Chinese preservice kindergarten teachers: The mediating role of work volition and career adaptability. *Front. Psychol.* 12:729504. 10.3389/fpsyg.2021.729504 35140645 PMC8818944

